# Supplementary material for: Chromothripsis during telomere crisis is independent of NHEJ, and consistent with a replicative origin
Source: Genome Res. 2019 May;29(5):737–49. doi: 10.1101/gr.240705.118 (PMC6499312; doi:10.1101/gr.240705.118)
Supplement: Supplemental Material [file supp_gr.240705.118_Supplemental_file_1.zip › contigs/annotated_contigs/DB38/contig.2.DB38_length_813_mean_cov_7.0110701107.docx]

**DB38_length_813_mean_cov_7.0110701107**

```
CCTTACAGCTCAATCAATTAACAATGGAAACGTAATTTTGGAAAGTATGGCCATCTCATTTAAAGAAACAAATTGCAAGCACTTTAGAA
 >chr17:34210337-34210710 + E=2e-212
ACATTCATCCAACCACACAAAAATGTTCTACCAATTGTTTCCTTCACAAAGCCCCGTTAGGTCTAAGGTAAGTTACCAAACATGTGAGA

GGTTCTGGAAAAGGCGGTGGGTTAAGAGCAGGGACTCGGCAGTTTCTCCTGTTTATTGTCCAGGTGGCCTCAGGTAAATTATTAATAAT

CTCTCTGGTCTCAGTTTCCTTACCTATAAATCAGTGATAATAATAATAATAGTTCCTCACACAACTATCGAGACAATTAAGTCCCTATA

AATGTTCTCATGCTTGC|T|CACTGCACCATGCTGGGCTAATTTTTAAATTTGTCGTATAGATGGGATCTTGCTATGTTGCCCAGGCTG
                   >chr17:34212093-34212532 + E=3e-246
GTCTCGAACTCCTGGCTTCAAGCAATCCTCCCACCTTGGCCTCCCAAAGTGCTGGGGTTGTAGGCATGAACCGCCACACCCAGCCATGG

GAGTATTTTTCTTCCCTTTCAGTGATTCTAATGGATTGTGAGGAGCACAGGAGGGAGGAGAATCTAATTTTTATTTCATGTCTACCATT

CATTTTCTCCTGTGCAAAATCAAGGTTTGGGCTTGATGGGCTATCTTCAATTTTTTCTCTGGTGCTAATGAACTGATTCCATAAGCAAA

TTAGAGAGCCCATGTAAACAGTATGGTGCCTGGTACAGGGTGGGTGTTCACTGAATGGCAGCTATCATTATTATTCACCCAGCACATTA

TGTTCCTGAAAACC
```
